# Mortality, complications and long-term functional outcome in elderly patients with fragility fractures of the acetabulum

**DOI:** 10.1186/s12877-020-1471-x

**Published:** 2020-02-17

**Authors:** Johannes Wollmerstädt, Philipp Pieroh, Isabell Schneider, Suzanne Zeidler, Andreas Höch, Christoph Josten, Georg Osterhoff

**Affiliations:** Department of Orthopaedics, Trauma and Plastic Surgery, University Hospital Leipzig, 04103 Leipzig, Germany

**Keywords:** Geriatric, Acetabulum, Mortality, Functional outcome, Non-operative, Complications, Fracture

## Abstract

**Background:**

Early operative treatment of acetabulum fractures in geriatric patients has been suggested to reduce pain and allow for earlier mobilization. The aim of this study was to determine mortality, complications and functional outcome after operative and non-operative treatment.

**Methods:**

Patients aged ≥60 years with operative treatment of low-energy fragility fracture of the acetabulum from 2009 to 2016 and a follow-up of at least 24 months were identified. The patients were contacted by phone and a modified Merle d’Aubigné score was obtained. If patients or their relatives were not available for follow-up, mortality data was assessed using a national social insurance database.

**Results:**

One hundred seventy-six patients (mean age 78, SD 10 years; 73 female) were available for analysis of mortality data. At final follow-up (68 months, SD 26, range, 24 to 129), 99/176 patients (56.3%) had deceased. One-year-mortality was 25.0% and 2-year mortality 35.8%. Type of treatment (non-operative vs. operative) did not affect mortality at 1 and 2 years (*p* = .65 and *p* = .10). Hospital-acquired infections were observed in 31/176 cases (17.6%), thromboembolic events and delirium in 6 patients (3.4%). In-hospital mortality was 5.7%. Patients who underwent operative treatment were more likely to have an in-hospital infection (*p* = .02) but less likely to sustain thromboembolic events (*p* = .03).

The mean hospital stay was 14 days (SD 10 days, range, 1 to 66). Patients with operative treatment were longer hospitalized than patients with non-operative treatment (*p* < .001).

The rate of secondary conversions to THA was 12.4%, this was not affected by initial treatment.

The mean modified Merle d’Aubigné Score of those patients available for a final follow-up (*n* = 47; follow-up 56 months, SD 28, range, 24 to 115) was 14/18 points, SD 3 (range 7 to 18). Functional results at final follow-up between operatively and non-operatively treated patients were without difference.

**Conclusions:**

All-cause mortality and in-hospital complications are high among geriatric patients with low-energy fractures of the acetabulum even when treated operatively. Secondary conversion rates to THA are similar to those seen in younger patients. Mid-term functional outcome in those surviving is fair.

## Background

Over the past decades andwith an ageing population, fragility fractures of the acetabulum have become more common and thus a greater concern [1].

A nationwide study covering the population of Finland, reported an increase by 30% in the incidence of acetabular fractures in individuals aged older than 65 within the past 20 years, with the incidence now being 23/100,000 per year [2]. Ferguson et al. even found a more than twofold increase amongst North American patients beyond the age of 60 within a similar time period [3].

Whereas the main mechanism of injury for younger patients is high-energy-trauma, the majority of acetabular fractures in the elderly are a result of a fall from standing height [2–5].

Like in geriatric fractures of the hip, the very similar impact of acetabular fractures on walking ability has the potential of high morbidity and mortality due to immobilization and complications associated with bedrest.

The treatment of displaced acetabular fractures in younger patients usually consists of open reduction and internal fixation, but since most of the elderly patients present with various comorbidities and decreased bone quality, an individualized treatment approach is necessary. Furthermore, patients of greater age are in need of early mobilization with regard to these comorbidities, and usually cannot follow partial weight-bearing protocols [6]. Treatment may vary from non-operative treatment, open reduction and internal fixation, minimally invasive percutaneous screw fixation to even primary total arthroplasty of the hip [7, 8].

It has been suggested, that operative treatment of these fractures may reduce pain, allow for earlier mobilization and shorter hospitalization time, thus leading to less associated complications [8, 9]. In contrast, as suggested by a smaller cohort study, operative treatment may also lead to more conversions to total hip arthroplasty [10].

It remains unclear, whether these potential short-term benefits also translate into better functional outcome and a lower mortality rate and would accordingly justify the perioperative risks of a surgical intervention.

The aim of this study was therefore to assess mid- to long-term mortality and functional outcome after geriatric acetabular fractures and to compare the outcome after surgical and conservative treatment in elderly patients.

## Methods

### Patients

The study protocol of this study was approved by the institutional ethics committee. Consecutive patients aged ≥ 60 years who had operative or conservative treatment of a low-energy fracture of the acetabulum between January 2008 and December 2016 with a follow-up of at least 24 months were identified from a prospective database of patients with acetabular fractures. The database is kept at our institution as part of the German Pelvis Registry and includes epidemiologic data and data on fracture patterns, therapy and in-hospital complications. For all patients identified from this database, a retrospective chart review was performed and eligibility was assessed. Exclusion criteria included the presence of an additional fracture of the lower extremity or pelvis and pathological fractures. Also, patients who had expressed objection to the use of their data for research purposes were excluded.

By chart review, additional information on comorbidities (expressed as ASA - American Society of Anesthesiologists Scale), complications and revision surgery was obtained. Mortality data was assessed using the national statutory pension insurances database. Patients, eligible according to the inclusion and exclusion criteria, were contacted by phone and after acquiring informed consent, functional outcome was assessed by the modified Merle d’Aubigné Score [11]. If the patients could not be contacted by a first phone call, two more attempts were made by phone and mail. Informed consents were acquired by phone and archived as mp3 audio files.

If patients were not able to understand the Merle d’Aubigné questionnaire due to language or cognitive impairment, they were excluded from the functional outcome assessment. In case personal contact could not be established, family doctors were contacted to assess mortality data and data on the conversion to total hip arthroplasty (THA).

### Outcome

Primary outcome was the all-cause mortality rate at 1 and 2 years. Survival was assessed through the national social insurance database (Deutsche Rentenversicherung) that provides mortality data for every German resident who received statutory pension.

Secondary outcomes were in-hospital complications, duration of the hospitalization, and secondary conversion to THA. In those patients who had survived the follow-up, functional outcome was assessed by a modified Merle d’Aubigné Score through telephone interviews.

### Statistical analysis

Statistical analysis was performed with SPSS for windows 25.0 (SPSS, Chicago, Illinois, USA). Data are presented as frequencies (n) and means with range and standard deviation (SD). To assess differences in means between different treatment groups, an independent-samples t-test was used for the normally distributed continuous data and a Chi-Square or Fisher’s exact test for categorical data. Missing data were reported as such for each outcome parameter. The level of statistical significance was set at *p* < 0.05.

## Results

**Fig. 1:**
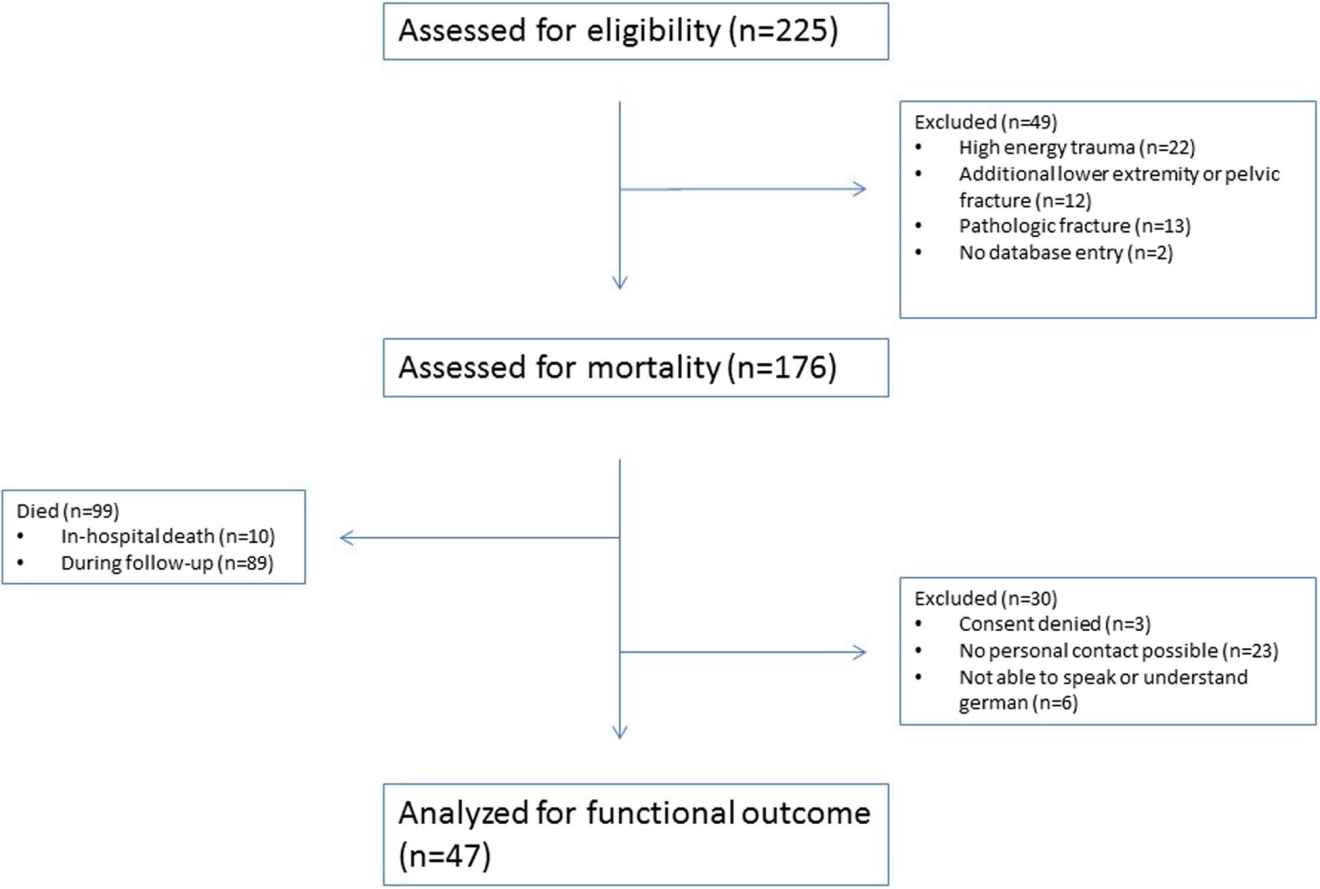
Patient flow chart

One hundred seventy-six patients (*n* = 176; mean age 78, SD 10 years; 73 female) were available for final analysis of mortality data (Fig. 1). Of those who had survived at the time of data acquisition (*n* = 77), 47 patients were available for functional outcome assessment. Mean ASA-Score (American Society of Anesthesiologists Scale) was 2.8 ± 0.5. Patients who were treated non-operatively were more likely to have an ASA score > 2 (56/67, 83.6%) when compared to patients who were treated operatively (75/109, 68.8%, *p* = .03).

**Fig. 2:**
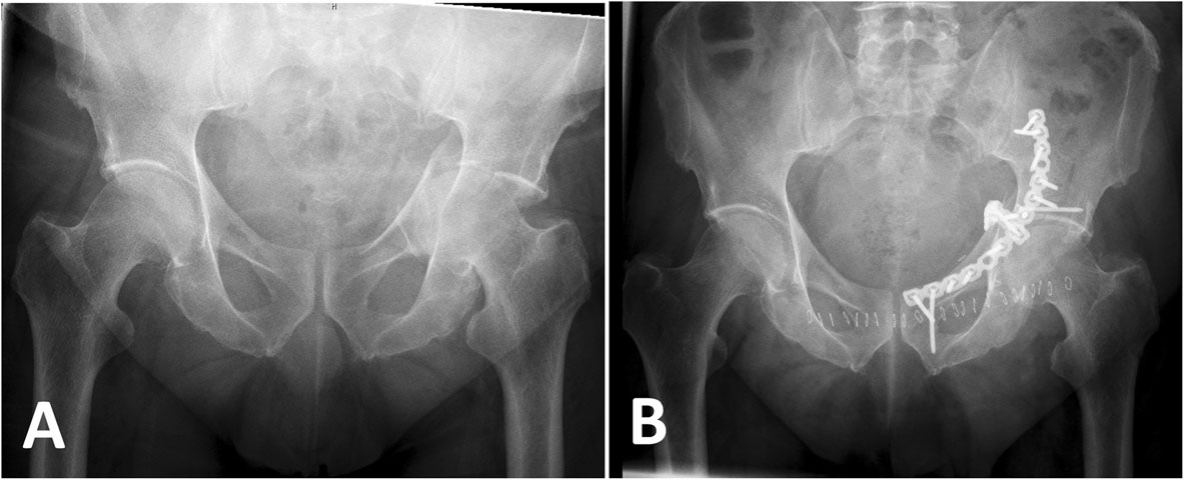
Case. Seventy-five year-old male patient with a left acetabulum fracture after a fall from standing height. **a** Pelvis AP radiograph on admission showing the central dislocation of the femoral head into the lesser pelvis. **b** Pelvis AP radiograph after open reduction and internal plate fixation

In 15 patients, the acetabular fracture occurred in the presence of a total hip implant in terms of a periprosthetic fracture. Of the remaining 161 patients with fractures of the native acetabulum, 67/161 patients (41.6%) were treated non-operatively, 10 patients (6.2%) by percutaneous fixation, 82 patients by open fixation (Fig. 2), and two patients (1.1%) received THA as primary treatment.

At the time of final follow up (68 months, SD 26, range, 24 to 129), 99/176 patients (56.3%) had deceased. Forty-four patients deceased during the first year after the hospitalization (1-year-mortality: 25%) and 2-year mortality was 35.8% (63/176 patients). The type of treatment (non-operative vs. operative) did not affect mortality at 1 and 2 years (*p* = .65 and *p* = .10, Table 1).

**Table 1:** Patients’ baseline characteristics, complications and survival (*N* = 176)

	Non-operative	Operative	
N	67	109	
Follow-Up [m]	70, SD 31	67, SD 27	.505^a^
Age [y]	80, SD 10	77, SD 9	.051^a^
Gender [f: m]	38: 29	35: 74	.002^b^
ASA			.033^c^
1	2	1	
2	9	33	
3	56	72	
4	0	3	
Hospitalization duration [d]	8, SD 6	17, SD 10	<.001^a^
In-hospital complications [n (%)]			
Infection	6 (9.0%)	25 (22.9%)	.024^b^
Thromboembolic event	5 (7.5%)	1 (0.9%)	.030^b^
Delirium	1 (1.5%	5 (4.6%)	.410^b^
In-hospital death	4 (6.0%)	6 (5.5%)	1.00^b^
1-year mortality	18 (26.9%)	26 (23.9%)	.654^b^
2-year mortality	29 (43.3%)	34 (31.2%)	.104^b^

^a^Student’s T-test. ^b^Pearson Chi-square/Fisher’s test

^c^Pearson Chi-square test for ASA ≤ 2 vs. ASA > 2. There were no patients with ASA > 4

Complications during the hospitalization occurred in 41/176 patients (23.2%). Hospital-acquired infections like surgical-site infections, pneumonia and urinary tract infections were observed in 31/176 cases (17.6%), thromboembolic events and a delirium were documented each in 6 patients (3.4%). Ten patients (5.7%) died during prolonged hospitalizations secondary to pneumonia (*n* = 4), pulmonary embolism (*n* = 3), myocardial infarction (*n* = 1), and delirium (n = 1). Patients who underwent operative treatment were more likely to have an in-hospital infection (*p* = .02) but less likely to sustain thromboembolic events (*p* = .03, Table 1).

The mean duration of the hospitalization was 14 days (SD 10 days, range, 1 to 66). Prior to the acetabulum fracture, 143/176 patients (81.3) had lived at home (43 with home care). After the fracture, only 93 (65.0%) of these 143 patients were able to eventually return to their homes (31 with home care). Patients with operative treatment stayed longer in the hospital than patients with non-operative treatment (*p* < .001, Table 1).

Twenty of the 161 patients (12.4%) with a non-periprosthetic fracture of the native acetabulum had to undergo secondary conversion to THA for posttraumatic osteoarthritis during follow-up. In the non-operative group, this was necessary in 5/67 patients (7.5%) versus in 15/92 patients (16.3%) in the operative group (*p* = .33).

The mean modified Merle d’Aubigné Score of those patients who had survived and were available for a final follow-up by phone (*n* = 47; follow-up 56 months, SD 28, range, 24 to 115) was 14/18 points, SD 3, range 7 to 18). Functional results at final follow-up were not different between survivors who had been treated operatively and non-operatively (Table 2).

**Table 2:** Functional outcome of survivors at final follow-up (*N* = 47)

	Non-operative	Operative	*p*
N	12	35	
Merle d’Aubigné	14, SD 3	15, SD 3	.890^a^
Pain	5, SD 1	5, SD 1	.748^a^
Mobility	5, SD 2	5, SD 1	.628^a^
Walking	4, SD 2	5, SD 2	.763^a^

^a^Student’s T-test

## Discussion

The aim of this study was to evaluate mortality and functional outcome of patients with geriatric fracture of the acetabulum, more than 2 years after treatment.

It was shown, that in this cohort of geriatric patients with fragility fractures of the acetabulum, all-cause mortality and in-hospital complications were high, regardless whether the patients underwent non-operative or operative treatment. While operatively treated patients were more susceptible to infection, thromboembolic events occurred more often in the non-operatively treated group. The conversion-rate to secondary total hip arthroplasty was 12.4%, with no significant difference between the groups. Patients surviving until final follow-up showed fair functional outcome and no difference between operative versus non-operative treatment occurred. We found no differences between the operative and the non-operative group in regards to all-cause 1-year-mortality. This finding is consistent with a previous studies that conclude that there is no difference in 1-year mortality between operative and non-operative management, once comorbidities are taken into account [4, 10].

Existing literature states 1-year mortality rates of 16 to 35% for non-operative treatment [10, 12–14] and of 4 to 21% for operative treatment [10, 12, 13]. Reasons for the higher mortality in the present study include the fact that a follow-up of 100% was achieved for mortality data by the use of the national social insurance database. In addition, only patients with a low-energy trauma were included. Patients older than 60 years of age with acetabular fracture as a result of high energy accidents are possibly in a better state of general health and live a more active lifestyle, than patients whose acetabular fracture is a result of same level falling and thus a representation of comorbidity.

The average duration of hospital stay was long (14 days, SD 10 days), compared to other studies that state a median hospitalisation of 8 to 11 days [10, 14, 15].Operatively treated patients had a longer average hospitalisation time, which is consistent with the findings of one previous publication on this topic [15].

The 47 patients that had survived until final follow up in the present study showed a fair functional outcome and no difference between operatively and non-operatively treated patients. While there seems to be consensus that good functional outcomes can be achieved by operative treatment of acetabular fractures in the elderly [12, 16–18], there is little data available on functional outcome after non-operative treatment. A recent retrospective case series of elderly patients with acetabular fractures that met criteria for surgery found that these patients can have good functional outcome also with non-operative treatment [14].

Secondary conversion rate to THA was 12.4% in the whole cohort and there was no significant difference between the operative and the non-operative group. Secondary conversion rates to THA have been reported to range from 10 to 37% [16] for patients in this age group when treated by open reduction and internal fixation and 15% when treated non-operatively [14]. A recent registry analysis of 678 acetabular fractures among all age groups reported that 19.8% of the patients required secondary THA [19]. Hence, the conversion rates found in our cohort are comparable to what is found in the literature.

The limitations of this study are inherent with its retrospective design. Therefore, selection bias for operative treatment is possible, since fractures with a less complex morphology were more likely to be assigned to non-operative treatment. Patients in the non-operative group had a higher ASA-Score, illustrating that these patients were in a worse state of general health and less amenable for both, primary and secondary operative interventions. No physical follow-up examinations were performed for functional outcome assessment. However, conducting follow-up interviews by phone enabled us to also assess patients, that were not able to leave their homes or nursing facilities and otherwise could not have been included. Still, we were able to only assess the functional outcome of 47 of 77 surviving patients resulting in a loss to follow-up of 39%. This comes with no surprise with regard to the geriatric population of this study and a follow-up interval of up to 10 years (129 months). Functional outcome was assessed mean 68 months after the trauma and in a small sample of patients who had mainly been treated operatively. This may provide a selection bias towards patients with better function resulting in better survival.

In conclusion, mortality and in-hospital complications remain high among geriatric patients with low-energy fractures of the acetabulum even when treated operatively. Secondary conversion rates to THA are similar to those seen in younger patients. Mid-term functional outcome in those surviving is fair.

Treatment decisions for these fractures in the elderly depend on multiple factors and can only be done individualized. The findings of this study may help surgeons in the decision-making and management of acetabular fractures after low-energy trauma in geriatric patients. The knowledge about the high rate of mortality and complications may guide surgeons towards non-operative treatment in very old patients with serious comorbidities. It may also be that selected elderly patients with acetabular fractures would benefit from primary THA as this usually allows for earlier mobilisation with full-weight bearing. Future prospective studies with larger cohorts need to further investigate the potential benefits of these different therapeutic strategies.

## Conclusions

All-cause mortality and in-hospital complications remain high among geriatric patients with low-energy fractures of the acetabulum even when treated operatively. Secondary conversion rates to THA are similar to those seen in younger patients. Mid-term functional outcome in those surviving is fair.

## Data Availability

Anonymized grouped data available upon request from the corresponding author.

## Abbreviations

ASA scale: American Society of Anesthesiologists Scale
SD: Standard deviation
THA: Total hip arthroplasty
